# Supplementary material for: Evaluating the health impact, health-system costs and cost-effectiveness of using TrueNat on stool samples compared to usual care for the diagnosis of paediatric tuberculosis in primary care settings: A modelling analysis
Source: PLOS Glob Public Health. 2025 Jun 26;5(6):e0004016. doi: 10.1371/journal.pgph.0004016 (PMC12200842; doi:10.1371/journal.pgph.0004016)
Supplement: S1 Appendix — (DOCX) [file pgph.0004016.s001.docx]

Evaluating the health impact, health-system costs and cost-effectiveness of using TrueNat compared to Xpert Ultra for the diagnosis of paediatric tuberculosis in routine settings: a modelling analysis.

Nyashadzaishe Mafirakureva^1†^, Olugbenga Kayode Daniel^2†^, Olabamiji Jamiu Olayinka^2^, Kingsley Chinedum Ochei^3^, Eveline Klinkenberg^4^, Austin Ihesie^3^, Debby Nongo^3^, Rupert Amanze Eneogu^3^, Andwele Mwansasu^5^, Emeka Uga Elom^6^, Agbaje Vivian Aderonke^2^, Patrick Sunday Dakum^2^, Charles Olalekan Mensah^2^, Oluwafemi Christopher Odola^2^, Abiola Oladotun Olayemi^2^, Emily Yemisi Faleye^7^, Adekunle Omotoso Makinde^8^, Peter J. Dodd^1^

1. Sheffield Centre for Health and Related Research, School of Medicine and Population Health, University of Sheffield, United Kingdom.
2. Institute of Human Virology, Nigeria.
3. USAID, Nigeria.
4. Independent consultant, Connect TB, The Hague, The Netherlands.
5. Infectious Disease Detection and Surveillance
6. Federal Ministry of Health, National Tuberculosis, Buruli Ulcer & Leprosy Control Programme, Nigeria
7. Osun State Ministry of Health, State Tuberculosis, Buruli Ulcer & Leprosy Control Programme, Osogbo, Nigeria
8. Oyo State Ministry of Health, State Tuberculosis, Buruli Ulcer & Leprosy Control Programme, Ibadan, Nigeria

^†^ Lead co-authors

# Patient care pathways

## Standard of care pathway

Care pathways for sick children (0-14 years) attending child healthcare services under the standard of care and intervention ([Figure S1](#ym29m2pnkn49) below) were developed based on reviewing relevant algorithms (Desk Guide for Prevention, Diagnosis and Treatment of Tuberculosis in Children and Adolescents in Nigeria) and discussion with IHVNigeria experts. Typically, children presenting at the primary health centre (PHC) or hospital level are screened for tuberculosis symptoms and those presumed to have tuberculosis are subject to further assessment for active tuberculosis disease ([Parameterization section](#_fhp0t6cgwm1c)). At PHC, a proportion of these children may be referred immediately without tuberculosis investigations (i.e in the presence of danger signs).

Children presumed to have tuberculosis undergo assessment for active tuberculosis disease. This may involve clinical assessment only with/without chest x-ray or clinical assessment with/without chest x-ray and bacteriological assessment depending on clinical need and test availability. Bacteriological assessment mainly involves sputum, stool, gastric or nasopharyngeal aspirate) on Xpert MTB/RIF (Cepheid, Sunnyvale, CA), hereinafter referred to as Xpert OR sputum only for TrueNat or TB LAMP test. Urine LF LAM assay may be used for eligible children with HIV. Generally, capacity for bacteriological assessment for children is limited at PHC (and some hospitals) due to unavailability of tests or inability of children to provide samples required for testing. Based on the assessment received, the outcomes can be diagnosis of tuberculosis (clinical or bacteriological) or no diagnosis. A proportion of children not initially receiving a tuberculosis diagnosis at PHC may be referred to a higher-level facility for further investigations and management. Children not meeting the criteria for tuberculosis diagnosis may be reassessed in 1-2 weeks if there are no improvements.

## Intervention pathway

The intervention was conceptualised as introducing TrueNat for bacteriological testing of children in PHC (or other) facilities where there is currently no access (direct or through sample referral) to bacteriological assessment using Xpert.

A 2019 study[1] on GeneXpert MTB/RIF performance reported that 23 (6.3%) of the 366 GeneXpert MTB/RIF machines uploading data during January–December 2017 were located in primary health care facilities. The study also reported a GeneXpert machine utilisation rate (Total tests performed/expected number of tests × 100) of 40% in PHC. Machines in primary health facilities had the highest (10.7%) proportion of unsuccessful test outcomes.

A study in 14 states of Nigeria found a low presence of TB diagnostic availability in PHC facilities – almost all states had <10% of their facilities with GeneXpert [2].


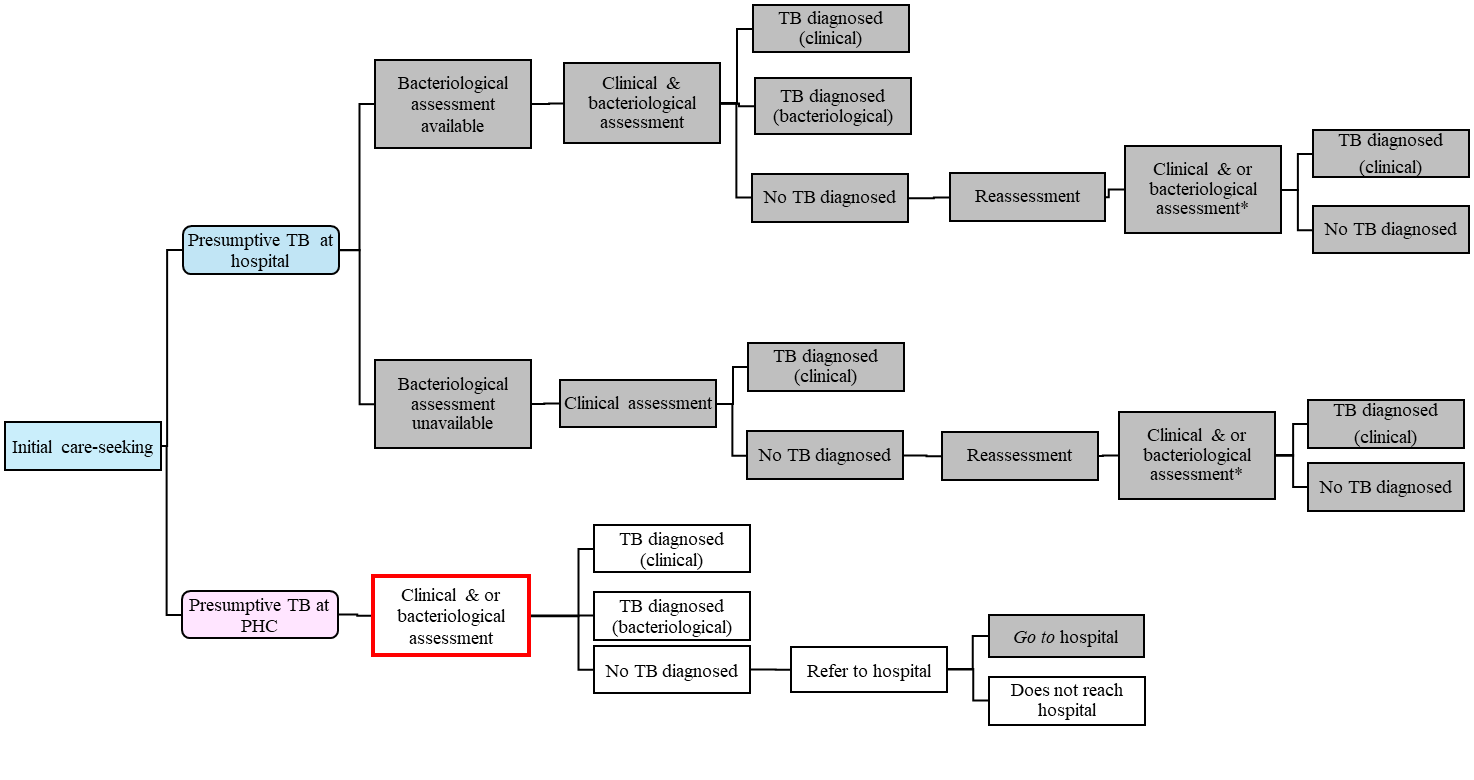


**Figure S1.** Simplified diagram showing the pathways of care for the management of children with symptoms suggestive of tuberculosis (presumptive TB). PHC=primary health centre, TB=tuberculosis. Boxes shaded in grey show activities undertaken at hospital level. Unshaded boxes show activities undertaken at PHC.

In addition to the limited availability of GeneXpert equipment, sub-optimal utilisation, interrupted power supply and challenges with specimen referral and transportation at PHC, children are often not able to produce the prerequisite specimen for testing. Under this background, primary modelling will assume no (very limited) availability of GeneXpert testing for children at PHC under the standard of care. The model schematic is meant to represent the pathways in a simplified way, but the actual model will be able to account for the different coverages of bacteriological test availability at both PHC and DH. Alternative coverage scenarios (e.g 0-10% coverage at PHC based on Odume et al.[2]) for access to GeneXpert testing under the standard of care can be explored in a sensitivity analysis.

## Pathway endings

Children diagnosed with active disease are initiated on anti-tuberculosis treatment using directly observed therapy at home or in the health facility. Selection of treatment regimen is based on disease severity, site, and drug susceptibility. Monitoring of anti-tuberculosis treatment depends on HIV status.

Where bacteriological testing (Xpert MTB/RIF Ultra or TrueNat) is undertaken, tuberculosis diagnosis may be of rifampicin-sensitive tuberculosis (RifS-TB) or rifampicin-resistant tuberculosis (RifR-TB). These results are used to select the appropriate treatment regime.

Where tuberculosis is diagnosed based on clinical examination (alone, or with CXR results) without positive Xpert results, tuberculosis is assumed to be RifS-TB and so RifS-TB treatment is given (which may be inappropriate due to RifR-TB being unrecognised).

Each branch ending in ‘No TB diagnosed’ in the main figure is followed by the ending presented in **Figure S2**.


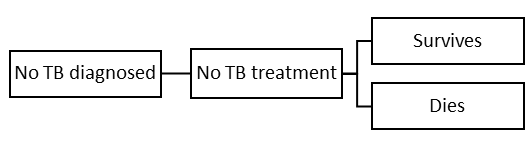


**Figure S2**. Branch ending in ‘No TB diagnosed’

Each branch ending in ‘TB diagnosed (clinical)’ in the main figure is followed by the ending presented in **Figure S3**.


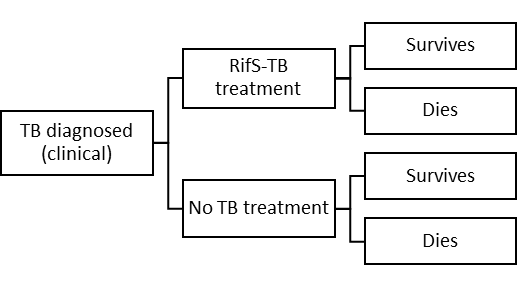


**Figure S3.** Branch ending in ‘TB diagnosed (clinical)’

Each branch ending in ‘TB* diagnosed (bacteriological)’ in the main figure is followed by the ending presented in **Figure S4**.


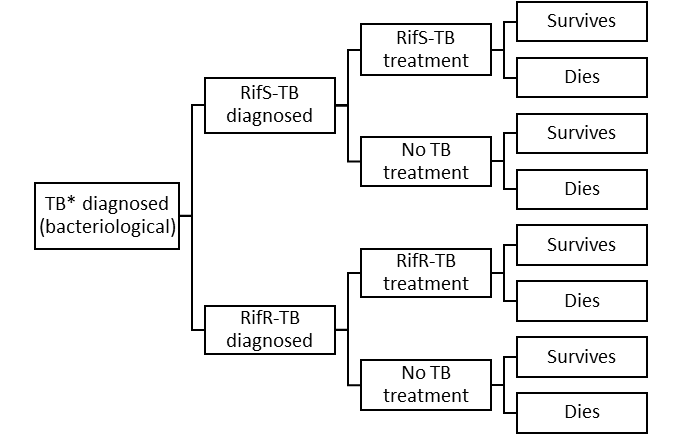


**Figure S4**. Branch ending in ‘TB* diagnosed (bacteriological)’


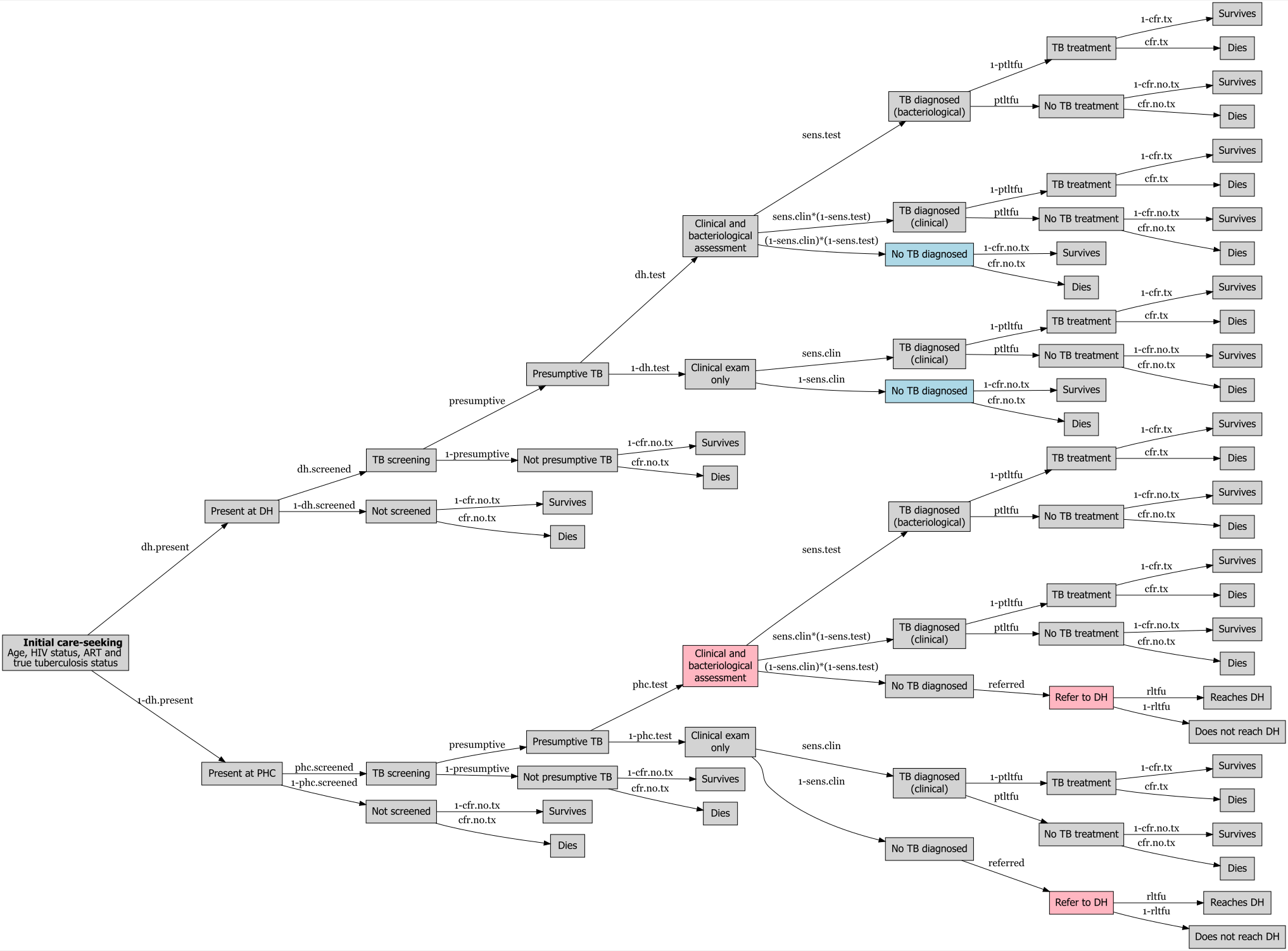


**Figure S5.** Simplified diagram of the decision-analytical tree model representing the pathways of care for TB diagnosis and treatment in children aged 0-14 years. The intervention has potential to impact on pink boxes by increasing the proportion of children receiving bacteriological assessment and reducing the proportion of children referred to higher level facilities for further assessments. Children in the ‘Reaches DH’ box enter the DH pathway. The pathway for children with ‘No TB diagnosed’ (the blue boxes) is simplified in this figure but may include reassessment after 2 weeks following which a TB diagnosis can be made. dh.present = proportion of children presenting to hospital level facilities, dh.screened/phc.screened = proportion of children screened for TB symptoms at DH/PHC, presumptive=proportion of children with presumptive TB, dh.test/phc.test=bacteriological test (Xpert Ultra or TrueNat) availability at DH/PHC, sens.clin/spec.clin=sensitivity/specificity of clinical diagnosis, sens.test/spec.test = sensitivity/specificity of bacteriological test (using sputum samples on Xpert Ultra or stool sample on TrueNat), ptltfu=pre-treatment lost to follow up, rltfu=referral lost to follow up, cfr.tx=case fatality rate for children on treatment, cfr.no.tx=case fatality rate for children not on treatment. ART; antiretroviral therapy, DH; district hospital, HIV; human immunodeficiency virus, primary healthcare; TB, tuberculosis; TB Tx, TB diagnosis and anti-TB treatment.

# Cost analysis

### Overview of cost analysis

The costs associated with each approach for the diagnosis and treatment of tuberculosis in children were estimated from a health service perspective: this considers all direct costs of resources required to deliver and implement each approach, regardless of payer. The analysis does not account for the patients’ direct non-medical costs (such as travel-related costs), and productivity costs (loss of earnings). The analysis followed three standard steps for conducting a cost analysis: identification of relevant resources based on the activities performed in each diagnostic approach; measurement of resources used; and applying relevant unit costs to value all the resources consumed by implementing each approach. The primary cost analysis focused on estimating the costs that differ between the two diagnostic approaches: i.e. the costs of the diagnostic tests. Downstream unit costs following a diagnosis were assumed to be similar and costs would only differ due different volumes of patients diagnosed and put on treatment. Costs were estimated and reported in 2024 United States dollar prices. All costs were assumed to accrue in the present, with no discounting applied.

### Resource use identification

This step lists all the relevant resources used to deliver each diagnostic approach, particularly those that differ between them. The main resources required are those associated with diagnosis and treatment of active tuberculosis disease. Relevant resources used to perform each test were identified by reviewing each test’s workflows, SOPs and discussions with the Nigeria TrueNat Stool Routine Implementation Pilot study team (hereinafter, the evaluation study). The TrueNat and Xpert sample processing and testing methods on stool involve different levels of infrastructural requirements, procedural complexity, numbers of steps, and material requirements which are necessary to capture. Given these potential differences, the main resources identified as relevant for the analysis included staff time, laboratory reagents and consumables, laboratory equipment and capital assets (i.e. medical and non-medical equipment), training, sample transportation, overhead (utilities, furniture, administrative, and operational costs) and building/infrastructure costs related to implementing the tests. Resources associated with treatment of children identified with tuberculosis were based on national tuberculosis program guidelines[^3^](https://www.zotero.org/google-docs/?xX85yV) and include use of outpatient (and possibly inpatient) care in addition to paediatric-specific anti-tuberculosis drugs. We assumed rifampicin-susceptible tuberculosis (RS-TB) treatment requires the standard 6-month regimen (2 months HRZE + 4 months HR). We assumed the following all-oral anti-tuberculosis drug regimens for children with MDR/RR-TB: children <5 years: 12 months Bdq-Lfx-Lzd-Cfz; children 5-15 years: 15 months Bdq-Lzd-Lfx-Cfz.

### Measurement of resource use

Information required to measure/quantify the resources used to perform the two diagnostic tests (Xpert and TrueNat) was collected from a sample of laboratory facilities participating in the evaluation study. The collected information is outlined for the different resource categories in the Table S1 below. Staff time spent performing the tests was measured directly using activity timing forms (time sheets) specifically designed for the evaluation study (attached separately). Activity timings data was collected from 6 TrueNat (30 timesheets) and 18 Xpert (81 timesheets) laboratories. Approximately 5 timesheets were completed for each laboratory. Laboratory staff activity timings are presented on Table S3. The rest of the data on Table S1 was collected from 5 TrueNat and 7 Xpert sites. Data collected included facility characteristics provided in Table S2. The use of cartridges, reagents and consumables was based on the actual quantities used to complete the TrueNat/Xpert test reported by the laboratories. These include the sample collection materials, sample processing materials and cartridges. Quantities of reagents and consumables were provided by the laboratories and validated by checking SOPs and discussions with key laboratory staff. Details of major laboratory equipment used to perform or support the tests were also provided by the laboratories. These included the type, number of pieces, useful life and annual usage (number of tests or active minutes). Information was also collected on staff training, sample transportation, building space, human resources and utilities.

**Table S1.** A list of key resources identified including details of how they were measured.

| **Type of resource** | **Information required** | **Sources of data** |
| --- | --- | --- |
| Direct staff time | - length of time spent on test activities - staff service hours - number and role(s) of staff involved in testing sample | - Staff timesheets/interviews - Laboratory records - Human resources |
| Cartridges, reagents and consumables | - quantity/volume of consumable supplies/kit for each test | - Laboratory records/manufacturer catalogue, SOPs |
| Sample collection materials | - quantity/volume used/required | - Laboratory records/manufacturer catalogue, SOPs |
| Major laboratory equipment | - number of pieces per items - annual number of uses - expected useful life | - Laboratory reports - Manufacture catalogues, purchase records |
| Staff training | - length of time spent on training - number of training events | - Laboratory records |
| Sample transportation | - number of samples - % non-TB samples | - Laboratory reports |
| Building space | - total facility space - size of laboratory space - number of staff (facility & lab) | - Laboratory/facility reports |
| Human resources | - total facility staff numbers - number of laboratory staff | - Laboratory reports/interviews |
| Laboratory characteristics including | - location (urban or rural) laboratory type (microscopy centre, reference laboratory etc) - population covered - operation hours - TB diagnostic tests (and other tests) available - annual number of TB tests/patients tested | - Laboratory reports/interviews |

**Table S2.** Characteristics of the 12 laboratory facilities where costing data was collected.

| **Test category** | **Location** | **Laboratory description** | **Tests performed** | **Service hours per day** | **Population coverage** | **Annual TB tests per patient** |
| --- | --- | --- | --- | --- | --- | --- |
| Xpert | Urban | GeneXpert and microscopic centre | Genexpert, TB LAMP and AFB Microscopy | 12 | 200273 | 5856 |
| Xpert | Urban | GeneXpert and microscopic centre | Genexpert, TB LAMP and AFB Microscopy | 10 | 128846 | 5028 |
| Xpert | Urban | Microscopy centre | Genexpert, TB LAMP and AFB Microscopy | 10 | 282585 | 4800 |
| Xpert | Rural | Microscopy centre | Genexpert, TB LAMP | 8 | 147600 | 2208 |
| TrueNat | Urban | Microscopy centre | TrueNat | 10 | 195874 | 672 |
| Xpert | Rural | Secondary health facility, Genexpert site | Genexpert, TB LAMP | 24 | 287460 | 9556 |
| Xpert | Urban | GeneXpert and microscopic centre | Genexpert, TB LAMP and AFB Microscopy | 9 | 796000 | 6200 |
| Xpert | Urban | Tertiary health facility, Genexpert Site | Genexpert | 8 | 796000 | 8581 |
| TrueNat | Urban | Microscopy centre | TrueNat | 10 | 240500 | 2148 |
| TrueNat | Rural | Primary health Centre, TrueNat Site | TrueNat | 9 | 180100 | 3150 |
| TrueNat | Rural | Primary health Centre, TrueNat Site | TrueNat assay and AFB microscopy | 9 | 102060 | 2520 |
| TrueNat | Rural | Primary health Centre, TrueNat Site | TrueNat | 8 | 174600 | 1200 |

### Valuation of resource use

Prices or cost data required to put monetary value on the resources used for the diagnostic tests identified and measured were collected from a sample of laboratory facilities participating in the evaluation study. Staff time spent performing the tests was valued using relevant monthly staff salaries. Costs/prices of reagents/consumables and equipment were based on costs provided by the laboratory facilities or public sources such as the Global Drug Facility if not provided. These were adjusted to reflect the number of possible uses (single versus multiple uses). The cost of laboratory equipment per minute was calculated by dividing the annualised cost of each piece of equipment by the total minutes the equipment was used in a year. A 3% annual discount rate was applied to annuitize equipment costs. The equipment cost per test was obtained by multiplying the cost per minute by the test duration reported by the sites. The cost of overhead, building expenses, non-laboratory staff salaries, utilities and administration per minute of operating the laboratory was estimated by dividing the total annual cost by the total annual operating hours. Sample transportation costs were based on the unit cost of $6 per sample, covering transportation of the sample to the laboratory and the results back to the health facilities. Unit costs for anti-tuberculosis treatment were derived from public sources using approaches previously described.[^4^](https://www.zotero.org/google-docs/?qd9yMv) Unit costs for anti-tuberculosis medicines were estimated using cost data available from the Global Drug Facility.[^5^](https://www.zotero.org/google-docs/?mR1nUb)

**Table S3**. Laboratory staff activity timings. Activity times presented as mean (standard deviation). DNA=deoxyribonucleic acid, MTB=mycobacterium tuberculosis, RIF=rifampicin.

| **Activity** | **Number of recordings** | **TrueNat** | **Xpert** |
| --- | --- | --- | --- |
| Receiving sample | 50 | 4.8 (1.6) | - |
| Sorting and pre-treatment | 50 | 10.1 (5.2) | - |
| Processing sample and initiating test: DNA extraction | 52 | 15.4 (7.9) | - |
| Processing sample and initiating test: DNA amplification | 52 | 12.5 (9) | - |
| Validation of MTB plus results | 51 | 11.3 (13.5) | - |
| Preparation for MTB-RIF assay | 6 | 10.3 (7.5) | - |
| Validating final results | 52 | 7.5 (8) | - |
| Recording and reporting results | 47 | 6.5 (4.9) | - |
| Receiving sample | 92 | - | 5.4 (10.8) |
| Sorting and pre-treatment | 92 | - | 8.9 (5.8) |
| Processing sample: technical preparation and initiating test | 92 | - | 15.5 (7.1) |
| Validating final results | 92 | - | 7.3 (6.3) |
| Recording and reporting results | 92 | - | 6.3 (4.1) |

### Total test costs

The total cost for each test was calculated as the sum of costs of equipment use, cartridges, reagents, consumables (sample and test), sample transportation, building space and overheads. The estimated unit cost for each contributing resource and the total cost per test are shown on Table S3 below.

**Table S4**. Estimated mean cost per test in 2024 United States dollars (US$).

| **Cost category** | **TrueNat** | **Xpert** |
| --- | --- | --- |
| Building space | 0.04 (0.03) | 0.04 (0.04) |
| Cartridges, reagents and consumables | 8.6 (0.02) | 10.52 (0.02) |
| Equipment | 2.87 (0.7) | 4.38 (1.31) |
| Human Resources | 0.68 (0.22) | 0.36 (0.12) |
| Sample collection materials | 0.43 (0.03) | 0.52 (0.03) |
| Sample transportation | 5.99 (0) | 5.99 (0) |
| Staff overheads | 0.42 (0.22) | 0.42 (0.24) |
| Utilities and administration | 0.02 (0.01) | 0.01 (0.01) |
| Total | 19.05 (1.45) | 22.24 (2.22) |

| 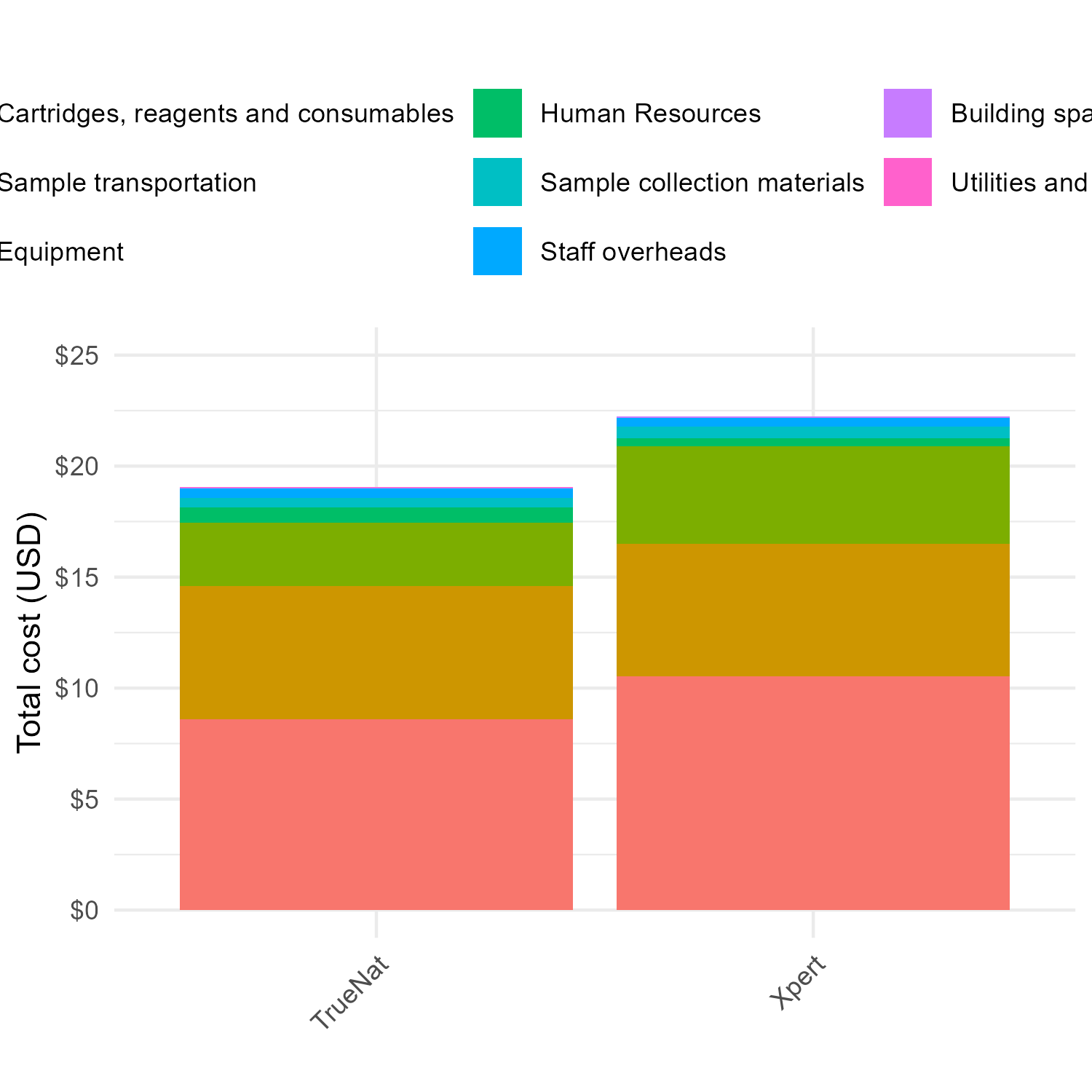 | 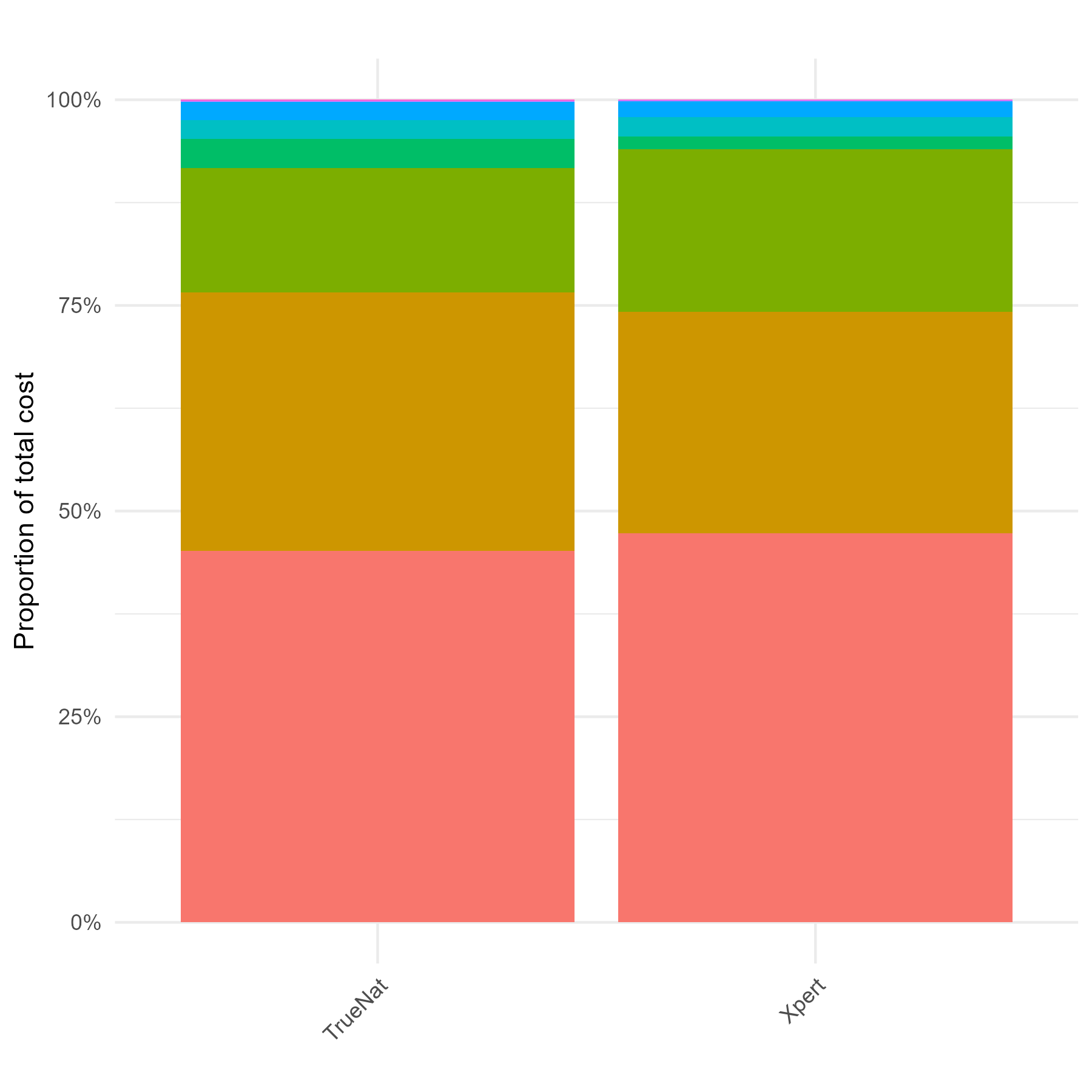 |
| --- | --- |

**Figure S6**. Distribution of mean costs per test by resource category

# Cost-effectiveness analysis

## Overview of model design and implementation

A decision analytic mathematical modelling approach was used to evaluate the health impacts, healthcare system costs, and cost-effectiveness of the intervention in comparison to the standard of care approach to the diagnosis of tuberculosis disease in children. The structure of a decision-tree model was based on the clinical care pathways shown in [Figure S1](#ym29m2pnkn49). The model was developed to capture the cascade of care for children presumed to have tuberculosis and were subject to further evaluation for active tuberculosis disease. Cascade steps before a child is presumed (presentation and screening for symptoms) to have tuberculosis were not explicitly modelled as these were assumed to remain the same under the intervention.

## Model parameters

The probability of children following different pathways through the tree was assumed to depend on these patient attributes: age (0–4 years or 5–14 years); HIV and antiretroviral treatment status (positive or negative); and true tuberculosis status (bacteriologically confirmed tuberculosis, bacteriologically unconfirmed tuberculosis, no tuberculosis). Bacteriologically confirmed tuberculosis is defined as tuberculosis that would be bacteriologically positive under ideal circumstances and with all samples available. Three main approaches were used to parameterize the decision tree model probabilities: data from past or ongoing studies in Nigeria, literature reviews and expert opinion/assumptions. As much as possible, parameters were based on empirical data from Nigerian studies or programme data. Available information from the scientific literature, from our previous work in other countries, and from expert experience were considered to determine the most appropriate values to use for these parameters in the modelling.

### Tuberculosis prevalence

The prevalence of true tuberculosis among children with presumptive TB was based on assumptions made in previous modelling work and depended on level of care (PHC and hospital level) [7]. This was assumed to be 25% (15% - 40%) at PHC and 50% (25% - 75%) at hospital level.

### Level of initial care-seeking

Primary data on characteristics of children (age and level of care where they initially present) was limited to Institute of Human Virology, Nigeria (IHVN) tuberculosis programmes and the Nigeria TrueNat Stool Routine Implementation Pilot. Data from the IHVN tuberculosis programme suggests that 36% of children presented to primary healthcare (PHC) facilities and slightly more than half (52%) were in the younger age group of 0-4 years. Following guidance from the patient-pathway analysis (PPA) methodology [5], we reviewed data from the 2018 Nigeria Demographic and Health Survey (2018 NDHS) [6] focussing on the place of care-seeking for children under 5 years old with fever in the two weeks prior to the DHS survey was used as a proxy for TB care-seeking. This data suggested that 82.1% of children initially sought care from PHC (primary care (Level 1) or informal private sector sites (Level 0)). A PPA of tuberculosis services in Cameroon reported that 87% initially sought care at Level 0 or 1 facilities [7]. Our previous analyses in similar settings assumed values of 90% (80-100%) [3,4]. Therefore, we used the NDHS estimate of 82% (72-92%) as the basecase (the 95% uncertainty interval was at +/- 10% points around the central estimate) and explored the impact of assuming different (lower/higher) values in a sensitivity analysis.

### Identification of presumptive cases of TB

All children presenting to healthcare facilities were assumed to be screened for tuberculosis symptoms. The proportion of children presumed to have tuberculosis was informed by the diagnostic accuracy of symptoms (≥ 1 of cough, fever, or decreased playfulness) for screening of pulmonary tuberculosis in child contacts [8]. Sensitivity was used for tuberculosis presumption in children with true disease while specificity [69% (95% CI 51% to 83%)] was used for children with no tuberculosis disease. The proportion of children with no disease presumed to have tuberculosis was based on 1 minus the specificity. The intervention was not assumed to change these steps of the cascade hence these were kept similar for both standard of care and intervention.

### Initial diagnostic processes

Diagnosis is modelled as a function of proportion of facilities with access to bacteriological testing, proportion of bacteriological test type available (Xpert or TrueNat in this case) and proportion of children who can produce a sample that in reality can be tested using the available test.

#### Facilities with bacteriological testing

Availability of bacteriological tests (particularly GeneXpert) at primary care and hospital level was informed by data from Odume et al.[2] This was defined as the proportion of facilities with access to bacteriological testing for tuberculosis and was assumed to to be 0-10% at the primary healthcare level and 20-100% at the hospital level (based on Xpert availability reported in Odume et al.[2]).

#### Bacteriological tests conducted

We assumed hospital level and higher facilities with bacteriological testing capacity would be using 100% Xpert under both standard of care and intervention (none of these facilities were assumed to have TrueNat). We assumed a very low coverage of Xpert availability at PHC under the standard of care; 0-10% based on data from Odume et al. [2] Under the intervention, we assumed similar Xpert coverage as under the standard of care but an increased coverage of TrueNat matching that of Xpert at hospital level (20-100%). We explored the impact of assuming100% TrueNat coverage at PHC in a sensitivity analysis.

#### Spontaneous sputum possible

The proportion of children who can be tested in reality was modelled depending on the possibility of obtaining a suitable sample. This was assumed to be 29.1% (21.5 - 37.5) for 5-14 years and 2.4% (2.0 - 2.7) for 0-4 years at hospital, and 9.2% (6.3 - 12.9) for 5-14 years and 2.4% (2.0 - 2.7) for 0-4 years at PHC) for Xpert based on our previous analyses [3,4], and 1 for TrueNat (assuming every child can provide a stool sample).

#### Bacteriological test diagnostic accuracy

Diagnostic accuracy for Xpert Ultra on respiratory samples for pulmonary tuberculosis in children was informed by data from a systematic review (Kay et al.).[^8^](https://www.zotero.org/google-docs/?YaKqZO) Diagnostic accuracy data for TrueNat on stool samples for pulmonary tuberculosis in children is currently not available. Singh et al.[9] recently reported the diagnostic performance of TrueNat on respiratory samples in comparison to microbiological reference standard: sensitivity of 57.1 (48, 65.9) and specificity of 92 (89.2, 94.2). We are currently assuming this as a proxy for the accuracy of TrueNat on stool samples.

#### Clinical diagnosis

Diagnostic accuracy for clinical diagnosis of tuberculosis in children is based on Marais et al. 2006 [10]: sensitivity of 62.7% (0.615 - 0.639) and specificity of 90.1% (0.894 - 0.908).

### Reassessment at 7 days

Data to inform reassessment at 7 days was not available and we assumed a proportion of children not returning for reassessment after 7/14 days similar to rates assumed in previous studies (around 20% (0-40%) [3,4].

### Referral to hospital

Data on hospital referral lost to follow-up was not available and was assumed to be similar to rates assumed in previous studies (around 20% (0-40%) [3,4].

### Pre-treatment lost to follow-up

Pre-treatment lost to follow-up was based on IHVN tuberculosis programmes data which suggested that ~93-98% of children diagnosed with active tuberculosis disease initiated anti-tuberculosis treatment. We aimed for a pre-treatment lost to follow-up of ~5% (0-10%).

### Outcomes following active tuberculosis disease

Tuberculosis disease outcomes were modelled using previously described assumptions and approaches, [3,4,11] by applying published meta-analytic case fatality ratios specific to first-line treatment stratified by age, HIV and antiretroviral therapy status[12].

**Table S5**. Model parameters and their distributions

| **NAME** | **DESCRIPTION** | **DISTRIBUTION** | **MEAN (IQR)** | **SOURCE** |
| --- | --- | --- | --- | --- |
| ontxY | CFR children <5 years on TB treatment | LN( -3.963316,0.6457913) | 0.019 (0.012 - 0.029) | Jenkins et al 2017[12] |
| ontxO | CFR children 5-14 years on TB treatment | LN(-4.828314,0.4817445) | 0.008 (0.006 - 0.011) | Jenkins et al 2017[12] |
| hivartOR:mn | ORs of death on TB treatment, (OR HIV+ vs -) x (ART -/+): mean | MVN: [2.6375681, -0.5683867] |  | Jenkins et al 2017[12], Dodd et al 2017[11] |
| hivartOR:sg | ORs of death on TB treatment, (OR HIV+ vs -) x (ART -/+): variance | MVN: [[0.2325509,-0.2325509],[-0.2325509,0.6367345]] |  | Jenkins et al 2017[12], Dodd et al 2017[11] |
| notxY | CFR children <5 years without TB treatment | LN(-0.830113,0.08035318) | 0.436 (0.413 - 0.460) | Jenkins et al 2017[12] |
| notxO | CFR children 5-14 years without TB treatment | LN(-1.903809,0.1285165) | 0.149 (0.137 - 0.162) | Jenkins et al 2017[12] |
| notxHAY | CFR children <5 years without TB treatment (HIV+/ART+) | B(15.18683,12.87500) | 0.542 (0.478 - 0.605) | Dodd et al 2017[11] |
| notxHAO | CFR children 5-14 years without TB treatment (HIV+/ART+) | B(10.43383,11.08417) | 0.484 (0.412 - 0.558) | Dodd et al 2017[11] |
| hivpi | IRR for TB incidence given HIV+/ART- (for individuals) | LN(2.066863,0.2800718) | 7.900 (6.540 - 9.543) | Dodd et al 2017[11] |
| artp | HR for TB incidence given HIV+/ART+ vs HIV+/ART- | LN(-1.203973,0.150482) | 0.300 (0.271 - 0.332) | Dodd et al 2016[13] |
| HHhivprev04 | Prevalence of HIV in children aged 0 4 years | B(44.0506,1791.391) | 0.024 (0.018 - 0.032) | Odafe et al 2020[14] |
| HHhivprev514 | Prevalence of HIV in children aged 5 14 years | B(44.0506,1791.391) | 0.024 (0.018 - 0.032) | Odafe et al 2020[14] |
| artcov | ARTChildren aged 0 to 14 receiving ARTI per year | B(186.9637,457.7387) | 0.29% (0.25 - 0.32%) | UNAIDS[15] |
| sens.clin | Sensitivity of symptom screening for detecting active pulmonary childhood tuberculosis | B(472.6527,281.1794) | 0.627 (0.592 - 0.661) | Marais 2006[10] |
| spec.sympt.screen | Specificity of symptom screening for detecting active pulmonary childhood tuberculosis | B(9.64,21.5) | 0.89 (0.6 - 0.90) | Vonasek et al. 2021[8] |
| sens.sympt.screen | Sensitivity of clinical diagnosis without CXR | B(492.167344045368,292.788547574039) | 0.69 (0.51 - 0.83) | Vonasek et al. 2021[8] |
| spec.clin | Specificity of clinical diagnosis without CXR | B(667.0122,73.2899) | 0.901 (0.878 - 0.921) | Marais 2006 |
| sens.truenat.sputum | Sensitivity of Truenat MTB-Rif on respiratory samples in bac+ children | B(65.68906,49.55491) | 0.571 (0.48,0.659) | Singh et al. 2023[9] |
| spec.truenat.sputum | Specificity of Truenat MTB-Rif on respiratory samples in bac+ children | B(415.2759,36.11095) | 0.92 (0.892, 0.942) | Singh et al. 2023[9] |
| sens.xstool | Sensitivity of Xpert Ultra on stool in bac+ children | B(21.716911549042,13.5951397502133) | 0.617 (0.561 - 0.672) | Kay 2022[16] |
| spec.xstool | Specificity of Xpert Ultra stool in bac+ children | B(480.118305785123,7.31144628099173) | 0.986 (0.982 - 0.989) | Kay 2022[16] |
| sens.xsputum | Sensitivity for C+ of Xpert on sputum | B(103.163282735012,38.5445232196748) | 0.753 (0.643 - 0.838) | Kay 2022[16] |
| spec.xsputum | Specificity for C+ of Xpert on sputum | B(520.629938271604,13.3494855967078) | 0.971 (0.947 - 0.985) | Kay 2022[16] |
| dh.ptltfu | pre-treatment loss to follow up | B(3.6,68.4) | 0.046 (0.031 - 0.064) | 5% (0-10%). Assumption |
| phc.ptltfu | pre-treatment loss to follow up | B(3.6,68.4) | 0.046 (0.031 - 0.064) | 5% (0-10%). Assumption |
| soc.dh.test | SOC, bacteriological test availability at DH | B(8.33, 3.57) | 0.70 (0.20-1.00) | Odume et al., 2023[2] |
| soc.phc.test | SOC, bacteriological test availability at PHC | B(3.6,68.4) | 0.05 (0.00-0.20) | Odume et al., 2023[2] |
| int.dh.test | INT, bacteriological test availability at DH | B(8.33, 3.57) | 0.70 (0.20-1.00) | Odume et al., 2023[2] |
| int.phc.test | INT, bacteriological test availability at PHC | B(8.33, 3.57) | 0.70 (0.20-1.00) | Odume et al., 2023[2] |
| soc.dh.exp.sputum.o5 | SOC, at DH: children 5-14 years receiving Xpert Ultra testing [either sputum or GA], in those identified as having presumptive TB | B(4.48,10.45) | 0.291 (0.215 - 0.375) | d Elbée et al. 2024[4] |
| soc.dh.exp.sputum.u5 | SOC, at DH: children 0-4 years receiving Xpert Ultra testing [either sputum or GA], in those identified as having presumptive TB | B(21.572,877.28) | 0.024 (0.020 - 0.027) | Mafirakureva et al. 2021[3] |
| soc.phc.exp.sputum.o5 | SOC, at PHC: children 5-14 years receiving Xpert Ultra testing [on sputum], in those identified as having presumptive TB | B(3.5,31.5) | 0.092 (0.063 - 0.129) | d Elbée et al. 2024[4] |
| soc.phc.exp.sputum.u5 | SOC, at PHC: children 0-4 years receiving Xpert Ultra testing [on sputum], in those identified as having presumptive TB | B(21.572,877.28) | 0.024 (0.020 - 0.027) | Assumption |
| dh.14dhltfu | 20%: 7-day loss to follow up, patients invited back for reassessment 7 days later (having initially been assessed as not having TB) | B(2,8) |  | Assumption |
| soc.phc.rltfu | 20%: referral loss to follow up, people referred to DH from PHC | B(2,8) |  | Assumption |
| Fbc.u5 | Fraction of children bacteriologically confirmable 0-5 years | B(17,23) | 0.424 (0.371 - 0.477) | Assumption |
| Fbc.o5 | Fraction of children bacteriologically confirmable children 5-14 years | B(3,3) | 0.500 (0.359 - 0.641) | Assumption |
| phc.tbprev | Prevalence of true TB among children with presumptive TB @ PHC | B(11.2748,33.8244) | 0.25 (0.15 - 0.40) | d Elbée et al. 2024[4] |
| dh.tbprev | Prevalence of true TB among children with presumptive TB @ DH | B(7.1832,7.1832) | 0.50 (0.25 -0. 75) | d Elbée et al. 2024[4] |
| OR.dh.if.TB.o5 | OR for initial care-seeking at DH-level given true TB, 5-14 years | LN( 2.136442,0.697286) | 2.1 (0.1 - 10.1) | d Elbée et al. 2024[4] |
| OR.dh.if.TB.u5 | OR for initial care-seeking at DH-level given true TB, 0-4 years | LN( 0.2886818,0.9521088) | 10.8 (0.1 - 33.6) | d Elbée et al. 2024[4] |
| phc.presented | Fraction initially seeking care at PHC | B(30.2,3.36) | 0.82 (0.72-0.92) | NDHS 2018[6] |

# Supplementary results

## Age-specific basecase results

### 0-4 years

**Table S6.** Healthcare resource use, health outcomes, costs & cost-effectiveness of the intervention in comparison to standard of care for children aged 0-4 years. Data are presented as means and 95% uncertainty interval from probabilistic sensitivity analysis unless otherwise stated. All costs are presented in 2024 United States dollars (US$). The incremental cost-effectiveness ratio (ICER) is presented as US$ per discounted disability-adjusted life year (DALY) averted.

|  |  |  |  |
| --- | --- | --- | --- |
| **Quantity per 100 children with presumptive TB (unless stated)** | **Standard of care** | **Intervention** | **Increment** |
| **Health-care resource use** | | | |
| Assessments | 104 (87 - 119) | 90 (78 - 102) | -14 (-24 - -3) |
| Percent assessments at PHC† | 46 (35 - 56) | 70 (51 - 86) | 25 (12 - 38) |
| Bacteriological assessments | 26 (15 - 37) | 43 (34 - 50) | 17 (3 - 30) |
| Percent bacteriological assessments at PHC† | 9 (2 - 22) | 73 (46 - 91) | 63 (37 - 84) |
| Referrals to hospital | 26 (16 - 36) | 9 (2 - 17) | -18 (-27 - -8) |
| Anti-tuberculosis treatments (ATT) | 20 (16 - 25) | 21 (17 - 26) | 1 (0 - 3) |
| Percent ATT initiated at PHC† | 53 (34 - 66) | 76 (52 - 91) | 23 (12 - 34) |
| Percent of true TB receiving ATT† | 82 (75 - 87) | 82 (77 - 86) | 0 (-4 - 5) |
| Percent of ATT bacteriologically confirmed† | 2 (1 - 4) | 25 (13 - 36) | 23 (11 - 34) |
| Percent of ATT false-positive† | 41 (26 - 59) | 43 (29 - 61) | 3 (1 - 6) |
| **Health outcomes** | | | |
| Tuberculosis diagnoses | 21 (16 - 27) | 23 (18 - 28) | 1 (0 - 3) |
| Percent of TB at PHC† | 54 (35 - 67) | 77 (53 - 91) | 23 (12 - 33) |
| Percent of TB bacteriologically confirmed† | 2 (1 - 5) | 25 (14 - 36) | 23 (12 - 34) |
| Deaths | 15 (13 - 18) | 15 (12 - 18) | 0 (-1 - 0) |
| Life-years lost | 387 (317 - 464) | 377 (310 - 447) | -11 (-32 - 4) |
| **Health systems costs** | | | |
| Cost (2024 US$) | 4274 (3074 - 5719) | 5453 (4156 - 7262) | 1179 (429 - 2197) |
| **Cost-effectiveness analysis** | | | |
| ICER (Cost per DALY averted) |  |  | 112 |
| †Indicates percentages calculated using different denominators. | | | |

**Figure S7.** Cost-effectiveness acceptability curves for the intervention in comparison to the standard of care for children aged 0-4 years. The figure shows the probability that an intervention is cost-effective (y-axis) based on the proportion of simulations in which the comparison of the intervention to the standard of care falls below the cost-effectiveness threshold (y-axis).


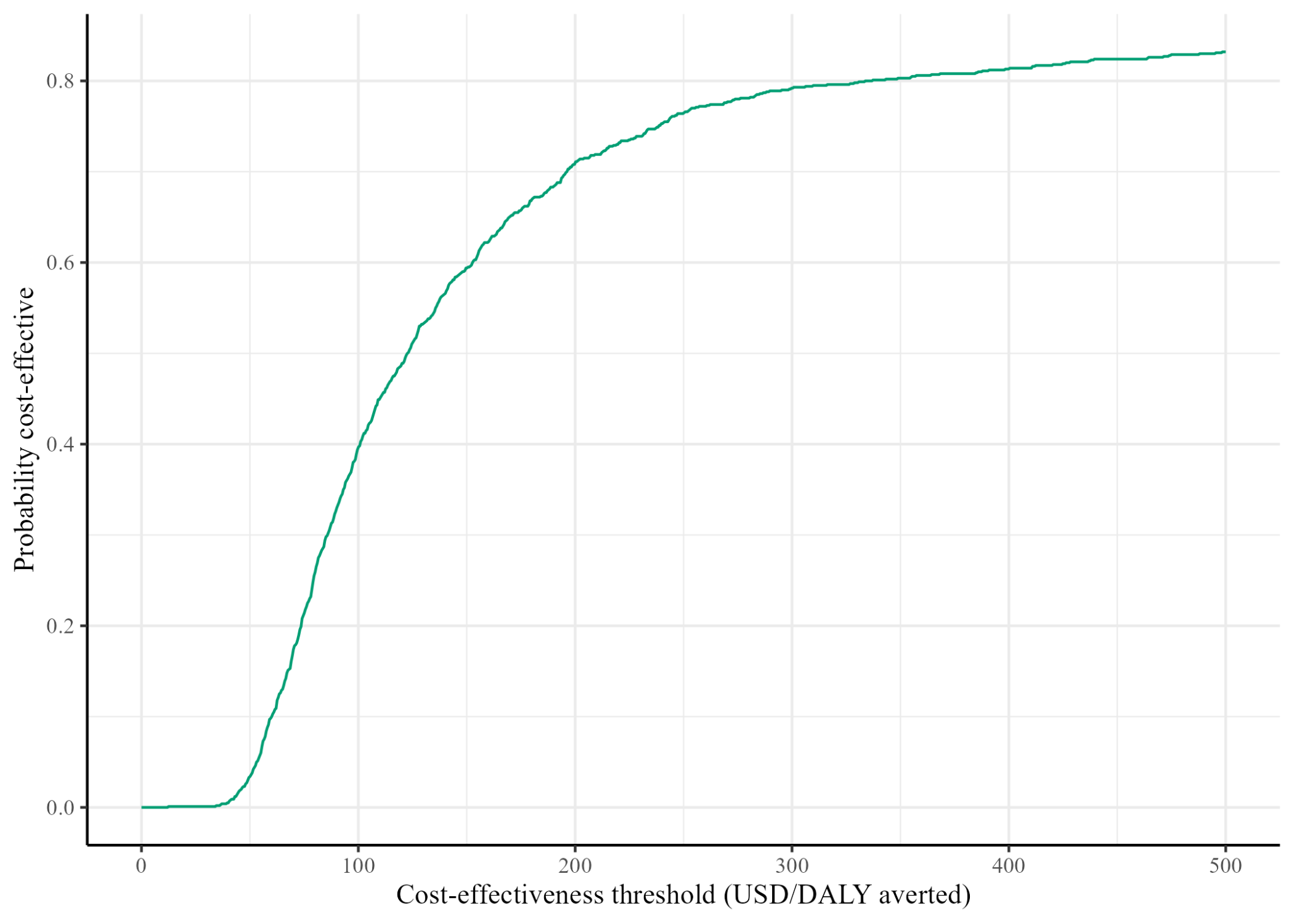


**Figure S8.** Cost-effectiveness plane showing the differences in costs (y-axis) and disability-adjusted life-years (DALYs, x-axis) of using the stool-based TrueNat for bacteriological testing using the simple one-step method for diagnosis of tuberculosis in children, compared with standard of care from 1000 simulations for children aged 0-4 years. Cost-effectiveness threshold (CET) lines show the cost-effectiveness (willingness to pay) threshold based on 1X GDP per capita (red line) or 0.5X GDP per capita (green line) in each country. The red dot represents the mean incremental costs and DALYs averted. ICER, incremental cost effectiveness ratio; CET, cost-effectiveness threshold; GDP, gross domestic product per capita, USD=United States dollar.


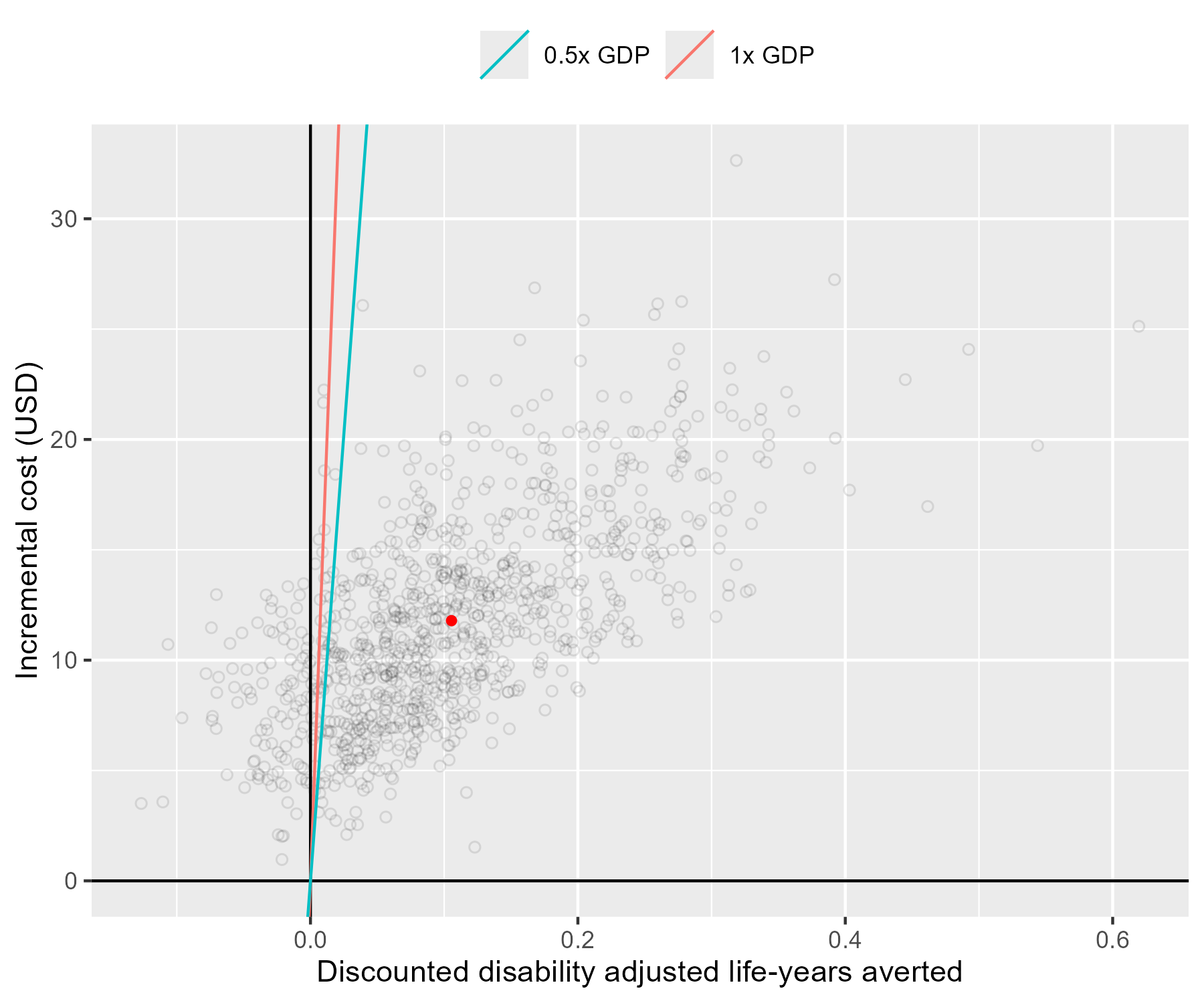


### 5-14 years

**Table S7.** Healthcare resource use, health outcomes, costs & cost-effectiveness of the intervention in comparison to standard of care for children aged 5-14 years. Data are presented as means and 95% uncertainty interval from probabilistic sensitivity analysis unless otherwise stated. All costs are presented in 2024 United States dollars (US$). The incremental cost-effectiveness ratio (ICER) is presented as US$ per discounted disability-adjusted life year (DALY) averted.

| **Quantity per 100 children with presumptive TB (unless stated)** | **Standard of care** | **Intervention** | **Increment** |
| --- | --- | --- | --- |
| **Health-care resource use** | | | |
| Assessments | 88 (74 - 101) | 77 (67 - 87) | -11 (-20 - -2) |
| Percent assessments at PHC† | 41 (30 - 53) | 64 (45 - 83) | 23 (11 - 36) |
| Bacteriological assessments | 24 (14 - 34) | 37 (29 - 43) | 13 (1 - 24) |
| Percent bacteriological assessments at PHC† | 8 (2 - 20) | 66 (39 - 88) | 58 (32 - 80) |
| Referrals to hospital | 21 (12 - 30) | 7 (2 - 14) | -14 (-22 - -6) |
| Anti-tuberculosis treatments (ATT) | 18 (14 - 22) | 19 (15 - 23) | 1 (0 - 3) |
| Percent ATT initiated at PHC† | 41 (22 - 59) | 62 (36 - 84) | 21 (10 - 31) |
| Percent of true TB receiving ATT† | 83 (77 - 89) | 84 (78 - 89) | 1 (-2 - 5) |
| Percent of ATT bacteriologically confirmed† | 6 (2 - 13) | 25 (14 - 39) | 19 (8 - 31) |
| Percent of ATT false-positive† | 41 (26 - 59) | 44 (29 - 62) | 3 (0 - 7) |
| **Health outcomes** | | | |
| Tuberculosis diagnoses | 18 (14 - 23) | 19 (15 - 24) | 1 (0 - 3) |
| Percent of TB at PHC† | 41 (21 - 59) | 61 (35 - 84) | 21 (10 - 32) |
| Percent of TB bacteriologically confirmed† | 7 (2 - 14) | 25 (14 - 39) | 19 (8 - 31) |
| Deaths | 5 (4 - 6) | 5 (3 - 6) | 0 (0 - 0) |
| Life-years lost | 119 (91 - 151) | 115 (88 - 146) | -4 (-10 - 0) |
| **Health systems costs** | | | |
| Cost (2024 US$) | 4469 (3261 - 6046) | 5966 (4452 - 7930) | 1498 (487 - 2772) |
| **Cost-effectiveness analysis** | | | |
| ICER (Cost per DALY averted) |  |  | 367 |
| †Indicates percentages calculated using different denominators. | | | |

**Figure S9.** Cost-effectiveness acceptability curves for the intervention in comparison to the standard of care for children aged 5-14 years. The figure shows the probability that an intervention is cost-effective (y-axis) based on the proportion of simulations in which the comparison of the intervention to the standard of care falls below the cost-effectiveness threshold (y-axis).


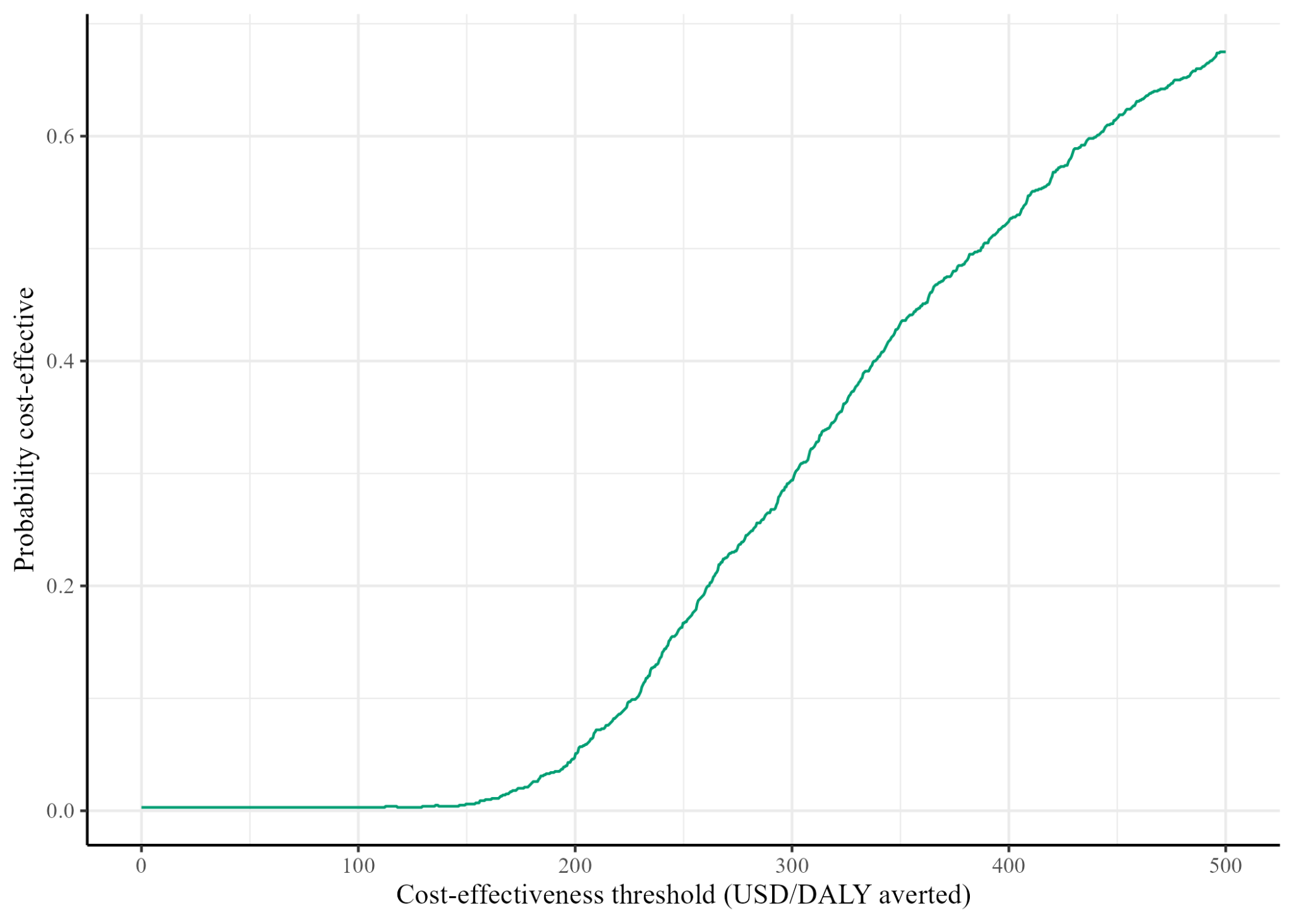


**Figure S10.** Cost-effectiveness plane showing the differences in costs (y-axis) and disability-adjusted life-years (DALYs, x-axis) of using the stool-based TrueNat for bacteriological testing using the simple one-step method for diagnosis of tuberculosis in children, compared with standard of care from 1000 simulations for children aged 5-14 years. Cost-effectiveness threshold (CET) lines show the cost-effectiveness (willingness to pay) threshold based on 1X GDP per capita (red line) or 0.5X GDP per capita (green line) in each country. The red dot represents the mean incremental costs and DALYs averted. ICER, incremental cost effectiveness ratio; CET, cost-effectiveness threshold; GDP, gross domestic product per capita, USD=United States dollar.


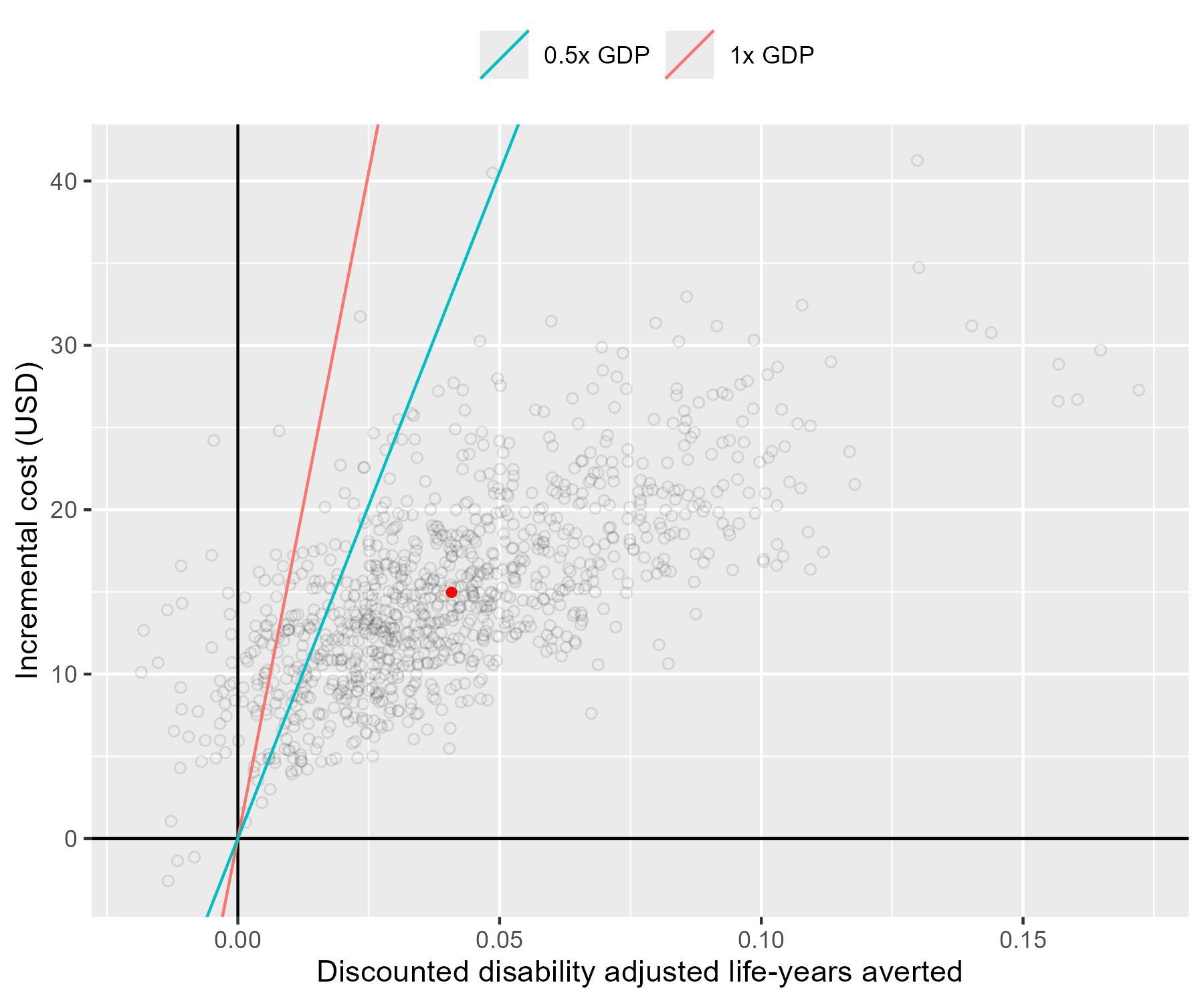


## Sensitivity analyses

### Impact of different assumptions

**Table S8.** The impact of different assumptions on the estimated incremental DALYs, costs and cost-effectiveness ratios. DALY=disability adjusted life year; ICER=incremental cost-effectiveness ratio

| **Assumption** | **DALYs averted** | **Incremental costs (2024 US$)** | **ICER (US$/DALY averted** |
| --- | --- | --- | --- |
| Basecase | -15 (-41 - 4) | 2682 (1039 - 4731) | 183 |
| 0% discount rate | -47 (-113 - -2) | 3166 (1295 - 5357) | 67 |
| 5% discount rate | -13 (-32 - -1) | 3166 (1295 - 5357) | 236 |
| Low PHC presentation | -4 (-14 - 2) | 676 (-462 - 1830) | 180 |
| Higher ART coverage | -20 (-48 - -1) | 3166 (1295 - 5357) | 158 |
| No baseline testing at PHC | -21 (-51 - 0) | 3331 (1337 - 5726) | 162 |
| Universal Truenat under intervention | -28 (-66 - 0) | 4540 (2357 - 7004) | 161 |

### Cost-effectiveness acceptability curves

**Figure S11.** Cost-effectiveness acceptability curves for the intervention in comparison to the standard of care for different modelling assumptions. The figure shows the probability that an intervention is cost-effective (y-axis) based on the proportion of simulations in which the comparison of the intervention to the standard of care falls below the cost-effectiveness threshold (y-axis). Base case refers to the analysis done with the most likely or preferred set of assumptions and input values. DALY=disability-adjusted life-years, USD=United States dollar


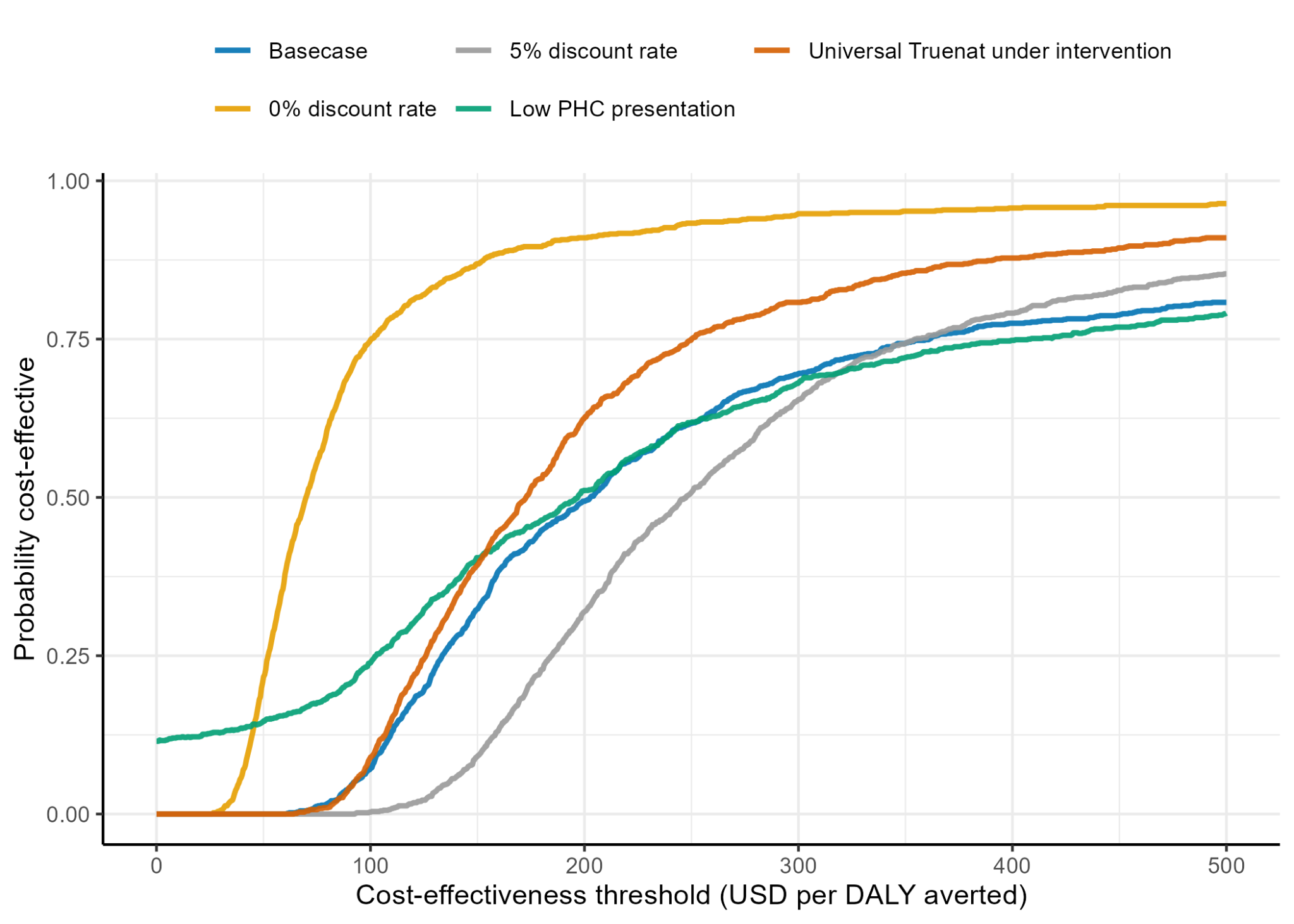


# References

1. Gidado M, Nwokoye N, Ogbudebe C, Nsa B, Nwadike P, Ajiboye P, et al. Assessment of GeneXpert MTB/RIF Performance by Type and Level of Health-Care Facilities in Nigeria. Niger Med J J Niger Med Assoc. 2019;60: 33–39. doi:10.4103/nmj.NMJ_12_19

2. Odume B, Useni S, Efo E, Dare D, Aniwada E, Nwokoye N, et al. Spatial Disparity in Availability of Tuberculosis Diagnostic Services Based on Sector and Level of Care in Nigeria. J Tuberc Res. 2023;11: 12–22. doi:10.4236/jtr.2023.111002

3. Mafirakureva N, Klinkenberg E, Spruijt I, Levy J, Shaweno D, de Haas P, et al. Xpert Ultra stool testing to diagnose tuberculosis in children in Ethiopia and Indonesia: a model-based cost-effectiveness analysis. BMJ Open. 2022;12: e058388. doi:10.1136/bmjopen-2021-058388

4. d’Elbée M, Harker M, Mafirakureva N, Nanfuka M, Nguyet MHTN, Taguebue J-V, et al. Cost-effectiveness and budget impact of decentralising childhood tuberculosis diagnosis in six high tuberculosis incidence countries: a mathematical modelling study. eClinicalMedicine. 2024;70. doi:10.1016/j.eclinm.2024.102528

5. Hanson CL, Osberg M, Brown J, Durham G, Chin DP. Conducting Patient-Pathway Analysis to Inform Programming of Tuberculosis Services: Methods. J Infect Dis. 2017;216: S679–S685. doi:10.1093/infdis/jix387

6. National Population Commission (NPC) [Nigeria] and ICF. Nigeria Demographic and Health Survey 2018 Key Indicators Report. Abuja, Nigeria, and Rockville, Maryland, USA: NPC, ICF; 2019 May. Available: http://ngfrepository.org.ng:8080/jspui/handle/123456789/3145

7. Titahong CN, Ayongwa GN, Waindim Y, Nguafack D, Kuate AK, Wandji IAG, et al. Patient-Pathway Analysis of Tuberculosis Services in Cameroon. Trop Med Infect Dis. 2021;6: 171. doi:10.3390/tropicalmed6040171

8. Vonasek B, Ness T, Takwoingi Y, Kay AW, Wyk SS, Ouellette L, et al. Screening tests for active pulmonary tuberculosis in children. Cochrane Database Syst Rev. 2021;2021: CD013693. doi:10.1002/14651858.CD013693.pub2

9. Singh UB, Singh M, Sharma S, Mahajan N, Bala K, Srivastav A, et al. Expedited diagnosis of pediatric tuberculosis using Truenat MTB-Rif Dx and GeneXpert MTB/RIF. Sci Rep. 2023;13: 6976. doi:10.1038/s41598-023-32810-2

10. Marais BJ, Gie RP, Hesseling AC, Schaaf HS, Lombard C, Enarson DA, et al. A refined symptom-based approach to diagnose pulmonary tuberculosis in children. Pediatrics. 2006;118: e1350-1359. doi:10.1542/peds.2006-0519

11. Dodd PJ, Yuen CM, Sismanidis C, Seddon JA, Jenkins HE. The global burden of tuberculosis mortality in children: a mathematical modelling study. Lancet Glob Health. 2017;5: e898–e906. doi:10.1016/S2214-109X(17)30289-9

12. Jenkins HE, Yuen CM, Rodriguez CA, Nathavitharana RR, McLaughlin MM, Donald P, et al. Mortality in children diagnosed with tuberculosis: a systematic review and meta-analysis. Lancet Infect Dis. 2017;17: 285–295. doi:10.1016/S1473-3099(16)30474-1

13. Dodd PJ, Prendergast AJ, Beecroft C, Kampmann B, Seddon JA. The impact of HIV and antiretroviral therapy on TB risk in children: a systematic review and meta-analysis. Thorax. 2017;72: 559–575. doi:10.1136/thoraxjnl-2016-209421

14. Odafe S, Onotu D, Fagbamigbe JO, Ene U, Rivadeneira E, Carpenter D, et al. Increasing pediatric HIV testing positivity rates through focused testing in high-yield points of service in health facilities—Nigeria, 2016-2017. PLOS ONE. 2020;15: e0234717. doi:10.1371/journal.pone.0234717

15. UNAIDS DATA 2024. Geneva: Joint United Nations Programme on HIV/AIDS. 2024 [cited 24 Jan 2025]. Available: https://www.unaids.org/en/regionscountries/countries/nigeria

16. Kay AW, Ness T, Verkuijl SE, Viney K, Brands A, Masini T, et al. Xpert MTB/RIF Ultra assay for tuberculosis disease and rifampicin resistance in children. Cochrane Database Syst Rev. 2022;9: CD013359. doi:10.1002/14651858.CD013359.pub3
