# Supplementary material for: Evaluating the health impact, health-system costs and cost-effectiveness of using TrueNat on stool samples compared to usual care for the diagnosis of paediatric tuberculosis in primary care settings: A modelling analysis
Source: PLOS Glob Public Health. 2025 Jun 26;5(6):e0004016. doi: 10.1371/journal.pgph.0004016 (PMC12200842; doi:10.1371/journal.pgph.0004016)
Supplement: Attachment — Submitted filename: Plos_Global_Public_Health_responses.docx [file pgph.0004016.s003.docx]

### Manuscript ID: PGPH-D-24-02630

Dear Dr Raquel Muñiz-Salazar,

We would like to thank you for the opportunity to revise our manuscript and the reviewers for the constructive feedback. We have carefully considered all the comments provided and have revised the text accordingly. In the enclosed document, we provide detailed responses to each of the reviewers’ points, outlining any specific changes made to the manuscript in response to their comments or suggestions.

As part of our investigation into Reviewer #2’s comments regarding the discrepancy between the increase in overall TB case detection and the increase in bacteriologically confirmed TB cases, we identified and corrected a bug in the model code that had caused this anomaly. This correction led to changes in the estimated increase in overall TB case detection (from 29 to 2 per 100 children with presumptive TB), bacteriological confirmation (from 6% to 21%), and the ICER (from $37 to $183 per DALY averted). However, the qualitative interpretation of the results remained unchanged.

## We appreciate your and the reviewers' time and effort in evaluating our work, and we hope these revisions meet your expectations.

## Thank you once again for your consideration.

Yours faithfully,

Dr Nyashadzaishe Mafirakureva, on behalf of all coauthors

KEY:

Reviewers' Comments & Our Responses:

Our responses are in blue.

We quote manuscript text:

*“In blue italics, with new or edited text underlined.”*

Response to reviewers’ comments

Reviewer #1: The manuscript is interesting, well-structured, and easy to follow. Here are some suggestions:
Clinical assessments are vital to TB diagnosis, and accurate clinical classification is necessary for subsequent evaluations. However, the model does not consider the proportion of cases that have the proper clinical classification.

Many thanks for taking the time to review the manuscript and for highlighting this important point. We are not completely sure what you mean here. The model assumes an underlying real TB status, and the performance of diagnostic tests (including clinical diagnosis) is defined and modelled with respect to this true status. In modelling the intervention, we have not assumed any change in the sensitivity and specificity of clinical diagnosis within the pathway. Apologies if this does not address your point - we are happy to respond again if you wish to clarify.

Some of the variables will not be assumed or taken from other countries. Example: The diagnostic accuracy of TruNat using stool might differ from that of TruNat using sputum; the loss to follow-up depends on the strength of the country's TB program, and data from other studies might not accurately represent Nigerian situations. The outcome of active TB considers age and HIV; nevertheless, this has to be taken from Nigeria.

Thank you for these points. We fully agree that country-specific parameters are crucial for modelling. However, this was often not feasible due to unavailability of relevant data. All the parameters in the model were discussed and agreed with in-country TB experts who deemed them suitable in the absence of local data. We also recognized the potential uncertainties associated with many parameters hence our decision to treat all parameters as uncertain in the model and our results reflect a range of possible parameter value sets. All assumptions are transparent, and their potential implications are discussed. Below, we provide more specific responses per parameter cited above.

The diagnostic accuracy of TruNat for paediatric tuberculosis diagnosis using stool samples has not yet been established. Therefore, we used diagnostic accuracy data for TruNat on respiratory samples. While we acknowledge the performance may differ in practice, current evidence for Xpert, a similar test, suggests comparable performance. Thus, we relied on data from sputum samples currently available. We highlighted this in the Methods section as follows:

*‘Diagnostic accuracy data for TrueNat on stool samples for pulmonary tuberculosis in children is currently not available. We assumed the recently reported diagnostic performance of TrueNat on respiratory samples in comparison to microbiological reference standard as a proxy for the accuracy of TrueNat on stool samples: sensitivity of 57.1 (48, 65.9) and specificity of 92 (89.2, 94.2).*[*^1^*](https://www.zotero.org/google-docs/?TfzSCq) *The performance of Xpert on stool has been shown to be comparable to that on sputum.*[*^2^*](https://www.zotero.org/google-docs/?Ut4Bf7)*’*

We also discussed this as a potential limitation of the analysis as follows:

*‘For example, diagnostic accuracy data for TrueNat specifically on stool samples for pulmonary tuberculosis in children is currently not yet available. In the absence of these data, we relied on recently reported diagnostic performance data on respiratory samples in comparison to microbiological reference standard*[*^1^*](https://www.zotero.org/google-docs/?lbJFp5) *as a proxy for the accuracy on stool samples. Diagnostic accuracy of Xpert on stool and respiratory samples has been shown to be comparable in a systematic review and meta-analysis focusing on children living in African countries.*[*^2^*](https://www.zotero.org/google-docs/?MuYKy3) *Assuming a lower accuracy for TrueNat on stool samples than what is reported for sputum samples could potentially reduce the cost-effectiveness of the intervention. Therefore, the analysis may require updating as new accuracy data becomes available.’*

We also completely agree that ‘the loss to follow-up depends on the strength of the country's TB program, and data from other studies might not accurately represent Nigerian situations’. However, due to the lack of country-specific data we assumed rates used in other studies in similar settings. Our estimate for this parameter aligns closely with pre-diagnosis loss-to-follow-up rates observed in similar settings, such as 28.3% in Uganda^1^ and 21% in Zimbabwe^2^. The 20% (0–40%) referral loss-to-follow-up estimate is conservative, as modelling a higher rate would improve cost-effectiveness by reducing cascade losses through fewer referrals from PHC to hospital.

1. Nuwematsiko R, Kiwanuka N, Wafula ST, *et al.* Pre-diagnosis and pre-treatment loss to follow-up and associated factors among patients with presumed tuberculosis and those diagnosed in Uganda. *BMC Health Serv Res* 2024; **24**: 1638.
2. Mando TC, Sandy C, Chadambuka A, *et al.* Tuberculosis cohort analysis in Zimbabwe: The need to strengthen patient follow-up throughout the tuberculosis care cascade. *PLoS One* 2023; **18**: e0293867.

*‘Data on referral loss to follow-up and 7/14-day loss to follow up was not available and was assumed to be similar to rates assumed in previous studies (around 20% (0-40%)).*[*^5,6^*](https://www.zotero.org/google-docs/?o70MiK)*’*

The age distribution of children modelled in the analysis was taken from Nigeria as stated in the manuscript:

*‘Age distribution of children was informed using data obtained from IHVN tuberculosis programmes and the Nigeria TrueNat Stool Routine Implementation Pilot.’*

We have now updated the HIV prevalence rate among children aged 0–14 years with estimates obtained from Nigeria. The data is based on facility-based HIV testing of 2,180 children seen at all facility points of service for the period 2016-2017.^1^ The proportion of children aged 0–14 years living with HIV (29% (25-32)) was obtained from UNAIDS data.^2^

“*Country-specific data on the prevalence of HIV among children aged 0–14 years seeking care in healthcare facilities (2.4% (1.8-3.2%)) was used in the model.*[^8^](https://www.zotero.org/google-docs/?bQYUFt) UNAIDS data for Nigeria,^9^ was used to inform antiretroviral coverage (29% (25-32)).

1. Odafe S, Onotu D, Fagbamigbe JO, *et al.* Increasing pediatric HIV testing positivity rates through focused testing in high-yield points of service in health facilities—Nigeria, 2016-2017. *PLOS ONE* 2020; **15**: e0234717.
2. UNAIDS DATA 2024. Geneva: Joint United Nations Programme on HIV/AIDS. 2024.<https://www.unaids.org/en/regionscountries/countries/nigeria> (accessed Jan 24, 2025).

We have also added the following text to highlight that all model parameters were discussed with and validated by in-country TB experts.

‘*The model structure and all the associated parameters were discussed and agreed upon with in-country TB experts.*’

The outcome variables are not clear, and please consider including definitions of terms. Examples: True TB, false TB, ATTs, etc.

Many thanks for this point. We have added the following definitions to help clarify these terms in the text.

*‘Anti-tuberculosis treatment (ATT) refers to the initiation of anti-TB drugs for individuals diagnosed with TB, whether confirmed or suspected; "true TB" refers to correctly diagnosed patients with TB, while "false TB" refers to those misdiagnosed with the disease despite not having it.’*

While the result in Table 2 indicates mean, the terms proportion and number are used interchangeably throughout the main text.

Many thanks for this point. In an effort to clarify this, we have revised the text defining the outcomes we calculated in the Methods section under the Heading ‘Health economic outcomes’ as follows:

“*For every 100 children seeking care with presumptive tuberculosis, we calculated the number of clinical assessments, bacteriological assessments, referrals, anti-tuberculosis treatments (ATTs), deaths, DALYs, and total costs, along with their changes under the intervention. Additionally, we calculated the percentage of true TB cases receiving ATT, ATT cases that are bacteriologically confirmed, ATT initiated at PHCs, and ATT cases that are false positives, as well as their changes under the intervention.*”

We have also clearly indicated the quantities presented as proportions in Table 2 using the dagger symbol (†).

*‘^†^Indicates percentages calculated using different denominators.’*

The result showed the intervention increased TB case detection by 29%, but the bacteriologically confirmed TB increased only by 6%. How does the implementation of stool-based TureNat testing increase TB case detection more, but it brings minimal change to the bacteriologically confirmed TB?

Many thanks for taking the time to review the manuscript and for picking up these discrepancies. As a result of investigating your comments, we identified and fixed a bug in the model code that resulted in the anomaly (see changes in GitHub repo). The updated model results are now consistent with the expected changes: the intervention increased TB case detection by 2 cases for every 100 children presenting with presumptive TB [from 39 (30 - 49) under SOC to 42 (33 - 52) under INT]. The percent of TB bacteriological confirmed increased by 21 (11 - 32) percentage points from 4% (2 - 8%) under SOC to 25% (15 - 36%) under INT.

We have updated Table 2 and the associated text & figures in the manuscript to reflect these changes.

Once more, our sincere thanks for your observation and question, which led us to identify this bug.

| **Quantity per 100 children with presumptive TB (unless stated)** | **Standard of care** | **Intervention** | **Increment** |
| --- | --- | --- | --- |
| **Health-care resource use** | | | |
| Prevalence of true TB | 28 (16 - 40) | 28 (16 - 40) | 0 (0 - 0) |
| Total assessments | 192 (160 - 220) | 167 (145 - 188) | -25 (-44 - -4) |
| Percent assessments at PHC† | 44 (33 - 54) | 68 (50 - 84) | 24 (11 - 38) |
| Bacteriological assessments | 50 (29 - 71) | 80 (63 - 93) | 30 (5 - 54) |
| Percent bacteriological assessments at PHC† | 8 (2 - 21) | 70 (43 - 89) | 61 (35 - 81) |
| Referrals to hospital | 48 (28 - 65) | 15 (4 - 32) | -32 (-50 - -14) |
| Anti-tuberculosis treatments (ATT) | 38 (29 - 47) | 40 (32 - 49) | 2 (0 - 6) |
| Percent ATT initiated at PHC† | 48 (32 - 61) | 70 (49 - 87) | 22 (11 - 32) |
| Percent of true TB receiving ATT† | 82 (76 - 88) | 83 (77 - 87) | 0 (-3 - 5) |
| Percent of ATT bacteriologically confirmed† | 4 (2 - 8) | 25 (15 - 36) | 21 (11 - 32) |
| Percent of ATT false-positive† | 41 (26 - 59) | 44 (29 - 61) | 3 (1 - 6) |
| **Health outcomes** | | | |
| Tuberculosis diagnoses | 39 (30 - 49) | 42 (33 - 52) | 2 (0 - 6) |
| Percent of TB at PHC† | 48 (32 - 62) | 70 (50 - 87) | 22 (11 - 32) |
| Percent of TB bacteriologically confirmed† | 4 (2 - 8) | 25 (15 - 36) | 21 (11 - 32) |
| Deaths | 20 (17 - 24) | 19 (16 - 23) | -1 (-2 - 0) |
| Disability-adjusted life years (DALYs) | 1184 (979 - 1406) | 1150 (956 - 1366) | -34 (-95 - 10) |
| Discounted DALYs | 507 (419 - 602) | 492 (409 - 585) | -15 (-41 - 4) |
| **Health systems costs** | | | |
| Cost (2024 US$) | 8731 (6720 - 11191) | 11414 (8910 - 14728) | 2682 (1039 - 4731) |
| **Cost-effectiveness analysis** | | | |
| ICER (Cost per DALY averted) |  |  | 183 |
| †Indicates percentages calculated using different denominators. | | | |

Reviewer #2: Overall, this is a well-written and informative manuscript. The research question is important and timely, and the study is well-designed. The authors have used a robust modelling approach to evaluate the potential impact of using TrueNat on stool samples for pediatric tuberculosis diagnosis, and their findings are likely to be of interest to policymakers and clinicians in Nigeria and other countries with a high burden of TB.

Here are some specific comments:
• Strengths:
o The study addresses an important and timely topic.
o The study design is appropriate for the research question.
o The authors have used a robust modelling approach.
o The study findings are likely to be of interest to policymakers and clinicians.

Many thanks for taking the time to review the manuscript and for these compliments.

Areas for improvement:
• Introduction:
o The introduction provides a good overview of the topic. However, it could be strengthened by providing more specific information on the burden of pediatric TB in Nigeria.

Many thanks for this suggestion. We have added some additional information on the burden of paediatric TB in Nigeria as follows:

*‘Nigeria bears one of the highest burdens of tuberculosis globally, ranking first in Africa and sixth worldwide, contributing approximately 4.6% of the global TB burden.*[^1^](https://www.zotero.org/google-docs/?h0mlVs) *In 2023, children aged 0–14 years accounted for 10% of the 367,250 TB cases reported (from an estimated incidence of 499,000) in Nigeria.*[^1^](https://www.zotero.org/google-docs/?aiRGtu) *However, TB notifications in children remain low, primarily due to under-diagnosis, especially among those under 5 years old. This is particularly concerning as TB tends to be more severe in children under 15, with the highest mortality observed in those under 5 years.*[^2^](https://www.zotero.org/google-docs/?s8lRcB)*’*

• Methods:
o The methods section is generally well-written. However, it could be improved by providing more detail on the decision tree model. For example, the authors could provide a figure of the decision tree.

Many thanks for this suggestion. Many thanks for this suggestion. As mentioned in the manuscript, the decision tree was coded based on the care pathways shown in Figure 1. A full description of the care pathway is given in the preceding sections on ‘Patient care pathways’. The full decision tree model after adding all the pathways and endpoints is very large and would not fit legibly on standard A4 pages. The reader would have to zoom in to see the boxes and labels, hence we didn’t think it was reasonable to include that in the paper. The full decision tree can be viewed by opening this [intervention CSV file](https://github.com/nmafirakureva/TruenatModel/blob/main/indata/CSV/INTc1.csv) and this [standard of care CSV file](https://github.com/nmafirakureva/TruenatModel/blob/main/indata/CSV/SOCc1.csv) in the GitHub repo as a spreadsheet. However, we have provided some additional text to help the reader better understand the modelling process.

“*The referral endpoints from PHC to higher level were modelled by adding an identical hospital care pathway to follow the two paths for referral from PHC level. This accounts for the flow of patients needing more specialised care or diagnostic services that cannot be provided at the PHC level. The intervention was modelled by replicating the entire pathway and increasing the proportion of children with access to bacteriological testing at PHC, enabled by the availability of TrueNat for stool samples.*’’

We have also added a less simplified decision tree in the Appendix (figure S5), with some explanation of the simplifications, and links to the full trees as CSV files given above.

• Results:
o The results section is clear and concise. However, it could be improved by providing more information on the uncertainty around the model estimates.

Many thanks for taking the time to review the manuscript and for these suggestions.

• Discussion:
o The discussion section is thoughtful and well-written. However, it could be improved by providing more specific recommendations for policymakers and clinicians.

Many thanks for this suggestion. We have added the following specific recommendations for policy makers where we think our analysis directly speaks to.

“*Stool-based TrueNat testing should be considered as an alternative approach for the diagnosis of pulmonary tuberculosis in children, particularly in routine healthcare settings where other bacteriological tests are not readily available. However, it is important to recognise that bacteriological testing, including TrueNat, can have a low negative predictive value, especially in young children. As such, the introduction of this technology should be accompanied by comprehensive training for healthcare professionals, ensuring that clinical assessment remains central to decision-making, particularly when negative test results are obtained.*

*Moreover, policymakers must carefully evaluate the full spectrum of costs associated with the implementation of TrueNat testing. This includes not only the procurement of equipment, reagents, and the establishment of supply chain logistics, but also the cost of training healthcare providers, developing or updating clinical guidelines, and improving infrastructure to support the integration of this technology. Additionally, consideration should be given to ongoing maintenance costs, the potential need for additional resources, and the long-term sustainability of such interventions within the healthcare system.*”

With these comments we have tried to provide policy makers with additional interpretation of our results, and suggestions of additional important considerations that could be relevant to decision making. We don’t feel that we should be more prescriptive than this in our role as analysts.

Specific comments:
• Page 3, line 11: You state that the intervention was cost-effective at a threshold of 0.5x GDP per capita. Please provide more information on how this threshold was chosen.

Many thanks for the comment. We have added some more information on how the threshold was chosen. Nigeria does not have an adopted threshold, and inferences based on econometric analyses are extremely uncertain. Our choice was intended to be illustrative and to anchor expectations without prescribing what local policy makers should be using as a threshold.

“*With no defined cost-effectiveness threshold for Nigeria, we assumed an illustrative threshold of 0.5×GDP per capita, aligning with recent reports suggesting thresholds lower than GDP per capita.^1^*[*^–3^*](https://www.zotero.org/google-docs/?OVYZ63) *ICERs falling below 0.5×GDP per capita suggest that the intervention is cost-effective against this threshold. However, the choice of threshold is ultimately a matter for policy makers.*”

1. Woods B, Revill P, Sculpher M, Claxton K. Country-Level Cost-Effectiveness Thresholds: Initial Estimates and the Need for Further Research. Value Health J Int Soc Pharmacoeconomics Outcomes Res 2016; 19: 929–35.
2. Ochalek J, Lomas J, Claxton K. Estimating health opportunity costs in low-income and middle-income countries: a novel approach and evidence from cross-country data. BMJ Glob Health 2018; 3: e000964.
3. Pichon-Riviere A, Drummond M, Palacios A, Garcia-Marti S, Augustovski F. Determining the efficiency path to universal health coverage: cost-effectiveness thresholds for 174 countries based on growth in life expectancy and health expenditures. Lancet Glob Health 2023; 11: e833–42.

• Page 4, line 21: You state that the intervention was implemented in PHC facilities where there was no access to bacteriological assessment using Xpert. Please provide more information on the selection criteria for these facilities.

Many thanks for the comment. For clarification, the intervention described in the analysis was not implemented in practice but was modelled based on a pilot implementation done in Nigeria during the first quarter of 2024. The quoted sentence is describing the conceptualisation of the intervention in relation to the existing standard of care.

‘*The intervention was conceptualised as introducing stool-based TrueNat for bacteriological testing using the SOS method of children in PHC facilities where there is currently no access to bacteriological assessment using Xpert directly or through sample referral. Under conditions of limited or no access to Xpert testing, the intervention would increase the fraction of children with a bacteriological test result at PHC and a reduction in referrals to higher healthcare level. Reduction in the need for referring children to higher level facilities would subsequently/possibly reduce referral lost-to-follow-up.* ‘

The hypothetical intervention was modelled as increased bacteriological testing coverage that matches current Xpert coverage at hospital level (20-100%) in the country. We have added some more details to further clarify this as follows.

*‘Availability of bacteriological tests (particularly Xpert) at primary care and hospital level was informed by discussions with country experts and coverage data from Odume et al.*[*^9^*](https://www.zotero.org/google-docs/?ZwmyfO) *Under the intervention, we assumed similar Xpert coverage as under the standard of care but an increased coverage of TrueNat at PHC matching that of Xpert at hospital level (20-100%).*[*^9^*](https://www.zotero.org/google-docs/?yY0vWY)*’*
